# Topical Dexamethasone Administration Impairs Protein Synthesis and Neuronal Regeneration in the Olfactory Epithelium

**DOI:** 10.3389/fnmol.2018.00050

**Published:** 2018-03-06

**Authors:** Umberto Crisafulli, André M. Xavier, Fabiana B. dos Santos, Tavane D. Cambiaghi, Seo Y. Chang, Marimélia Porcionatto, Beatriz A. Castilho, Bettina Malnic, Isaias Glezer

**Affiliations:** ^1^Department of Biochemistry, Escola Paulista de Medicina, Universidade Federal de São Paulo, São Paulo, Brazil; ^2^Department of Biochemistry, Instituto de Química, Universidade de São Paulo, São Paulo, Brazil; ^3^Department of Microbiology, Immunology and Parasitology, Escola Paulista de Medicina, Universidade Federal de São Paulo, São Paulo, Brazil

**Keywords:** anosmia, inflammation, corticoids, innate immune response, neurogenesis, neuronal cell death, S6 kinase, *Toll*-like receptor 4

## Abstract

Chronic inflammatory process in the nasal mucosa is correlated with poor smell perception. Over-activation of immune cells in the olfactory epithelium (OE) is generally associated with loss of olfactory function, and topical steroidal anti-inflammatory drugs have been largely used for treating such condition. Whether this therapeutic strategy could directly affect the regenerative process in the OE remains unclear. In this study, we show that nasal topical application of dexamethasone (DEX; 200 or 800 ng/nostril), a potent synthetic anti-inflammatory steroid, attenuates OE lesion caused by Gram-negative bacteria lipopolysaccharide (LPS) intranasal infusion. In contrast, repeated DEX (400 ng/nostril) local application after lesion establishment limited the regeneration of olfactory sensory neurons after injury promoted by LPS or methimazole. Remarkably, DEX effects were observed when the drug was infused as 3 consecutive days regimen. The anti-inflammatory drug does not induce OE progenitor cell death, however, disturbance in mammalian target of rapamycin downstream signaling pathway and impairment of protein synthesis were observed during the course of DEX treatment. In addition, *in vitro* studies conducted with OE neurospheres in the absence of an inflammatory environment showed that glucocorticoid receptor engagement directly reduces OE progenitor cells proliferation. Our results suggest that DEX can interfere with the intrinsic regenerative cellular mechanisms of the OE, raising concerns on the use of topical anti-inflammatory steroids as a risk factor for progressive olfactory function impairment.

## Introduction

Chronic rhinosinusitis is one of the most common inflammatory condition in the upper respiratory tract, a fact corroborated by the estimated proportion of affected individuals, 5–15% of the general population (Holbrook and Leopold, 2006; Bachert et al., 2014). Considering that roughly 14–30% of nasosinus disease patients present olfactory dysfunction (Holbrook and Leopold, 2006), it is possible to estimate that dozens of people in every thousand can present some degree of loss of sense of smell due to nasal mucosa chronic inflammation.

The OE, the tissue that transduces odorant signals into neuronal activity, is found on the nasal septum and on a series of turbinates in the posterior region of the nasal cavity (Mori and Sakano, 2011; Ihara et al., 2013). Mature OSNs are OE bipolar cells that transduce odorant signaling from the epithelial surface to the olfactory bulb of the brain (Graziadei and Monti-Graziadei, 1979). The OE is subjected to self-renewal throughout life due to the relatively short average life span of OSNs (Mackay-Sim and Kittel, 1991). Neuronal progenitors denominated globose cells (GBCs), which are located adjacent to the OE basal membrane, replenish the OSNs in a regular basis. However, horizontal basal cells (HBCs), the prototypical stem-cell of the OE, are recruited upon massive neuronal cell death in the OE in order to generate a large number of OSNs (Leung et al., 2007; Iwai et al., 2008), making the OE a remarkable site of adult neurogenesis (Brann and Firestein, 2014).

A significant decrease in quality of life is associated with loss of the sense of smell, including decreased appetite, depression, spoiled food consumption, and unawareness of potential danger signals such as smoke and exposure to dangerous chemicals; overall reducing psychological wellbeing (Blomqvist et al., 2004; Croy et al., 2014). It has been suggested that the extent of olfactory recovery depends on the duration of olfactory loss (London et al., 2008), implying that treatment strategies may impact the clinical outcome if the natural regenerative process is accelerated or delayed. Hence, it is a reasonable assumption that olfactory loss due to OE cells damage could be more or less permanent depending on the velocity of OSNs replenishment.

Acute models of OE lesion are valuable paradigms characterized by substantial and fast regeneration of OSNs, offering a straightforward procedure to evaluate if specific chemical compounds and biomolecules impair or enhance the neural cells replenishment. For instance, an analogous approach has been used for evaluating the impact of brain inflammation on oligodendrocyte repopulation after acute demyelination (Glezer et al., 2006; Franklin and Ffrench-Constant, 2008), and on CNS neurogenesis (Monje, 2003; Ekdahl et al., 2009). In this study, we show that engagement of innate immune response by bacterial LPS can be used as an inflammatory acute model of cell death in the OE. It is well characterized that LPS triggers TLR4 activation on the surface of myeloid cells, activating the transcription of several pro-inflammatory genes through nuclear factor kappa-B (NF-κB) and AP-1 transcription factors (Aderem and Ulevitch, 2000). GCs engage GRs and interfere at multiple levels with NF-κB/AP-1 pathway and the expression of pro-inflammatory genes. GCs have been successfully used for several decades for treating inflammatory diseases (Tsurufuji et al., 1979; Scheinman et al., 1995; Almawi et al., 1996; De Bosscher et al., 2003; Xavier et al., 2016). In fact, intranasal administration of GCs is considered one of the most effective treatment for rhinosinusitis and nasal polyposis (Avery, 1998; Meltzer et al., 2000; Parikh et al., 2001; Csomor et al., 2013; Fandino et al., 2013).

We first aimed to verify whether topically infused synthetic GC DEX, a strong and highly selective GR agonist devoid of mineralocorticoid effect (Rosewicz et al., 1988; De Bosscher et al., 2000), is able to protect the OE against acute inflammatory injury. Using an effective DEX dose compatible with the known therapeutic range, we investigated if the corticoid improves or interferes with OE regeneration and an associated mechanism. GCs have also been shown to interfere with protein synthesis (Wang et al., 2006; Shimizu et al., 2011) and neural stem cell proliferation (Samarasinghe et al., 2011). Mammalian target of rapamycin (mTOR) signaling pathway is a major positive modulator of both biological processes (reviewed in Russell et al., 2011; Saxton and Sabatini, 2017). In consequence, we also investigated whether GR signaling in OE affects mTOR pathway and protein synthesis during early events involved in olfactory regeneration at nasal mucosa level.

## Materials and Methods

### Animal Experimentation

Newborn (P1–P3) or 3 months old adult male C57Bl/6 mice (body weight, 25–29 g) from either *biotério do Conjunto das Químicas* (Universidade de São Paulo) and *Laboratório de Experimentação Animal – Instituto Nacional de Farmacologia e Biologia Molecular* (Universidade Federal de São Paulo) were acclimated to standard laboratory conditions (12/12 h light/dark cycle; lights on at 6:00 AM and off at 18:00 PM), with *ad libitum* access to rodent chow and water. This study was carried out in accordance with animal experimentation procedures approved by the local Committees (COBEA – USP, CEP – UNIFESP, and CEUA – UNIFESP).

### Intranasal Infusion

Mice were anesthetized under isoflurane gas and 10 μl of vehicle (sterile 0.9% saline) or LPS (O55:B5 Sigma–Aldrich^®^; 10 ng/μl) was infused with a PE-10 cannula (Braintree Scientific, Inc., MA, United States) by means of a microinjection pump with flow rate set to 4 μl/min. Intranasal infusions of thionine or bromophenol blue solutions in anesthetized animals, performed as terminal procedure, provided dye labeling used to optimize drug delivery and visualize solution distribution over the OE. For evaluation of anti-inflammatory effects, DEX (injectable dexamethasone-phosphate; ACHÉ Laboratórios, Brazil) was diluted to 20–80 ng/μl solutions in vehicle, which were infused as described above. Unless otherwise specified, only the right nostril was treated. For puromycin labeling of protein synthesis, animal received intranasal infusion of saline containing puromycin (100 ng/μl) 6 h after DEX topical application for each time-point. Labeling specificity was confirmed by CHX (10–1000 ng/μl) infusion 30 min in advance of puromycin infusion. For optimal puromycin labeling, the nostril was cleared with saline infusion prior to puromycin application. Rapamycin was infused at 50 ng/μl concentration.

### Systemic Methimazole Treatment Combined to Intranasal Infusion

The animals received either vehicle (sterile water) or methimazole (50 mg/kg) at the first and fourth day of experimentation as previously described (Bergman et al., 2002). This treatment causes total loss of OMP-positive OSNs and was found to be highly reproducible among animals. For the evaluation of DEX effects on OE regeneration, intranasal infusions of DEX (40 ng/μl) or vehicle were performed 24 h after the second injection of the methimazole, either as a single dose of DEX or for three consecutive doses every 24 h. Animals were subjected to euthanasia at time-points 1, 2, 3, or 14 days after the second injection of methimazole. In order to evaluate DEX effect in early time-points (1–3 days), animals were euthanized 6 h after the last intranasal infusion. For puromycin labeling, animals were euthanized 30 min after the antibiotic application. In case of experiments for immunoblotting, both nostrils received intranasal infusions.

### *In Vivo* Bromodeoxyuridine (BrdU) Labeling for Fate Mapping

Mice subjected to methimazole treatment (starting at experimental day 0) received i.p. injections of BrdU (50 mg/kg) two times per day (∼8:00 AM and ∼6:00 PM). BrdU injections started 72 h after the first methimazole administration (experimental day 3), and ceased (i.e., last injection) 3 days after the second methimazole administration (experimental day 6). Animals were euthanized 14 days after the second methimazole administration.

### Tissue Preparation

Animals were deeply anesthetized via an intraperitoneal injection of a mixture of ketamine hydrochloride and xylazine (300:30 mg/kg of ketamine/xylazine) and then rapidly perfused transcardially with 0.9% saline, followed by 4% formaldehyde in phosphate-buffered saline (PBS) pH 7.6, at 4°C. The noses were rapidly dissected, post-fixed, and decalcified for 2–4 weeks in 2% formaldehyde/0.25 M EDTA, and then placed in 20% sucrose complemented solution overnight at 4°C for cryoprotection. The nostrils were embedded in O.C.T. media and 16 μm coronal sections were cut on a cryostat (Microm). The slices were collected onto superfrost slides (Fisher Scientific), or equivalent, and stored at -20°C. A set of animals were perfused only with cold saline and OE was quickly dissected, snap-frozen, and stored at -80°C for immunoblotting.

### Immunoblotting

The tissue was homogenized in ice-cold lysis buffer (50 mM Tris–HCl pH 8, 150 mM NaCl, 1% NP-40, 0.5% sodium deoxycholate, 1 mM EDTA, 16 μg/ml benzamidine-HCl, 10 μg/ml phenanthroline, 10 μg/ml aprotinin, 10 μg/ml pepstatin A, 10 μg/ml leupeptin, 1 mM PMSF, 10 mM sodium fluoride, 1 mM sodium orthovanadate, 17.5 mM sodium beta-glycerophosphate, and 6 mM sodium pyrophosphate) with the aid of a microcentrifuge-compatible generator (Polytron^®^– Kinematica, Switzerland) to yield whole extracts. After debris clearing by centrifugation, protein concentration was determined using Bradford assay (Bio-Rad). The extracts were resolved in SDS–PAGE and transferred by electrophoresis onto PVDF membranes. For immunoblotting, membranes were blocked with 5% non-fat dry milk in Tween–Tris-buffered saline and then incubated in the same buffer with the following antibodies: anti-phospho-p70 S6 Kinase (Cell Signaling No. 9206), anti-PCNA (Abcam No. ab29), anti-OMP (WAKO No. 544-10001), and anti-β-actin (Millipore No. MABT523) or anti-eIF2alpha (Cell Signaling No. 5324) as loading controls. Membranes were washed with Tween–Tris-buffered saline six times for 5 min and then incubated with 1:10,000 secondary horseradish peroxidase-conjugated antibody (Jackson ImmunoResearch, Inc.) for 1 h. Following washing, immunoreactive bands were detected by SuperSignal^TM^ West Pico PLUS Chemiluminescent Substrate (Thermo Scientific No. 34580) and captured using a LumiBis DNR Bio-Imaging Systems device. Image densitometric analyses were performed with the aid of ImageJ software^1^.

### Neurosphere Generation and *in Vitro* Differentiation

Neurosphere preparation from rodent OE has been previously reported (Krolewski et al., 2011). Freshly dissected OE from eight newborn (1–3-day-old) mice were pooled and submitted to enzymatic dissociation with trypsin–EDTA 0.25% incubation for 10 min at 37°C. Trypsin was inactivated with 10% fetal bovine serum supplemented media and cells were mechanically dissociated with the aid of a micropipette. The suspensions were passed through a 40-μm cell strainer (Falcon^TM^) and centrifuged for 5 min at 143 × *g*. The supernatant was discarded and the pellet was suspended in DMEM/F12 supplemented with 1% penicillin, 1% streptomycin, 2% B-27 (Gibco^TM^), and 20 ng/ml of either EGF and FGF-2 (Sigma–Aldrich^®^). Viable cells were counted for plating at 2 × 10^5^ density in 24-well plates coated with poly-HEMA (Sigma–Aldrich^®^), and incubated with drugs at standard cell culture conditions (37°C, 5% CO_2_, and ∼85% humidity) for 6–7 days, when OE neurospheres were unequivocally identified in suspension. Spheroid counting was performed in 10 random microscope fields, and replicates of the same treatment were averaged. We only considered for analysis neurospheres with diameter >25 μm. DEX was used in concentrations ranging from 0.3 to 3 μg/ml, DEX–BSA was acquired from Steraloids (Newport, RI, United States) and used at equivalent molar concentration of DEX 1 μg/ml (∼2.12 μM), and mifepristone (RU486) was used at 2.55 μM.

In order to validate olfactory identity of the culture, OE neurospheres were differentiated into olfactory neurons. The spheroids were centrifuged for 5 min at 143 × *g*, resuspended in growth factor-deprived medium, and transferred onto 13 mm poly-lysine/laminin-coated coverslips. The neurospheres were allowed to differentiate in standard cell culture conditions for 7–14 days in the absence of growth factors, and subsequently differentiated cells were prepared for OMP immunostaining.

### Immunofluorescence

Phosphate-buffered saline-washed sections were blocked for 30 min with PBS containing 4% donkey serum, 1% BSA, and 0.4% Triton X-100. Using the same buffer solution composition, the sections were incubated for ∼16 h at 4°C with primary antibody (polyclonal goat anti-OMP, WAKO No. 544-10001; anti-Ki67, Abcam No. ab15580; and anti-puromycin, Millipore No. MABE343). OE sections were washed three times with PBS and primary antibody detection was conducted with compatible Alexa Fluor^®^-488 and -546 secondary antibodies (Molecular Probes^®^) in PBS/0.4% Triton^TM^ X-100/1% BSA for 90 min. Sections were then rinsed with PBS and counterstained with DAPI for nuclear labeling. For BrdU labeling, tissue sections were pretreated with HCl 2 M for 30 min at 37°C, washed several times, and incubated with mouse anti-BrdU conjugated with Alexa Fluor^®^-555 (Molecular Probes^®^). In the case of double-labeling of BrdU and OMP, both antibodies were incubated after HCl denaturation.

Differentiated neurospheres were washed with Dulbecco’s PBS and fixed with cold PBS-buffered formaldehyde 2% for 15 min. The coverslips were rinsed three times with PBS and blocked with PBS/0.1% Triton^TM^ X-100/10% horse serum for 1 h. The fixed material was incubated anti-OMP in blocking solution for 15 h at 4°C. After three PBS wash steps, secondary detection was performed with anti-goat conjugated with Alexa Fluor^®^-546 or -555.

### Terminal Deoxynucleotidyl Transferase dUTP Nick-End Labeling (TUNEL)

Tissue sections were labeled for cleaved DNA detection with *In Situ* Cell Death Detection Kit-TMR red (Roche) according to manufacturer instructions.

### Image Processing

Photomicrographs were acquired with epifluorescence microscopes (Nikon^®^ or Carl Zeiss) or confocal microscopes (Carl Zeiss or Leica). The images were processed to enhance contrast and image quality using GIMP^2^ and were assembled using Inkscape^3^. The image edition was kept to a minimum to avoid artifacts, and equivalent treatment was applied to pictures representing different groups.

### Image Analysis

For cell layer thickness measures, photomicrographs were analyzed directly with AxioVision (Carl Zeiss) image capture software. Uncalibrated Optical Densities (O.D.) measures of fluorescence signals were acquired with ImageJ^4^ or FIJI^5^. O.D. values were obtained subtracting background signal from non-stained tissue segments, and four different coronal sections were used per sample.

### Statistical Analysis

Comparison of group means was performed using a one-way ANOVA followed by a Tukey’s HSD procedure as *post hoc* multiple comparisons test. Student’s *t*-test was applied for comparison between two groups according to homogeneity of variances (Levene’s test). Statistical analysis was carried out with PSPP^6^ or STATISTICA 12 (StatSoft^®^) software. Analysis of neurosphere counting was performed with nonparametric methods in R (R Core Team, 2017), using Quade test for unreplicated complete block designs (each independent experiment was considered an independent block) and Quade *post hoc* test implemented in PMCMR package (Pohlert, 2014) with Holm multiple-testing procedure. Data are represented as mean ± standard error of the mean (SEM), except for neurosphere counting, which are represented as median ± interquartile range (IQR) and plotted with ggplot2 functions (Wickham, 2009).

## Results

### Topical DEX Co-infusion Prevents OE Lesions Triggered by a Single Bolus LPS Infusion

**FIGURE 1:**
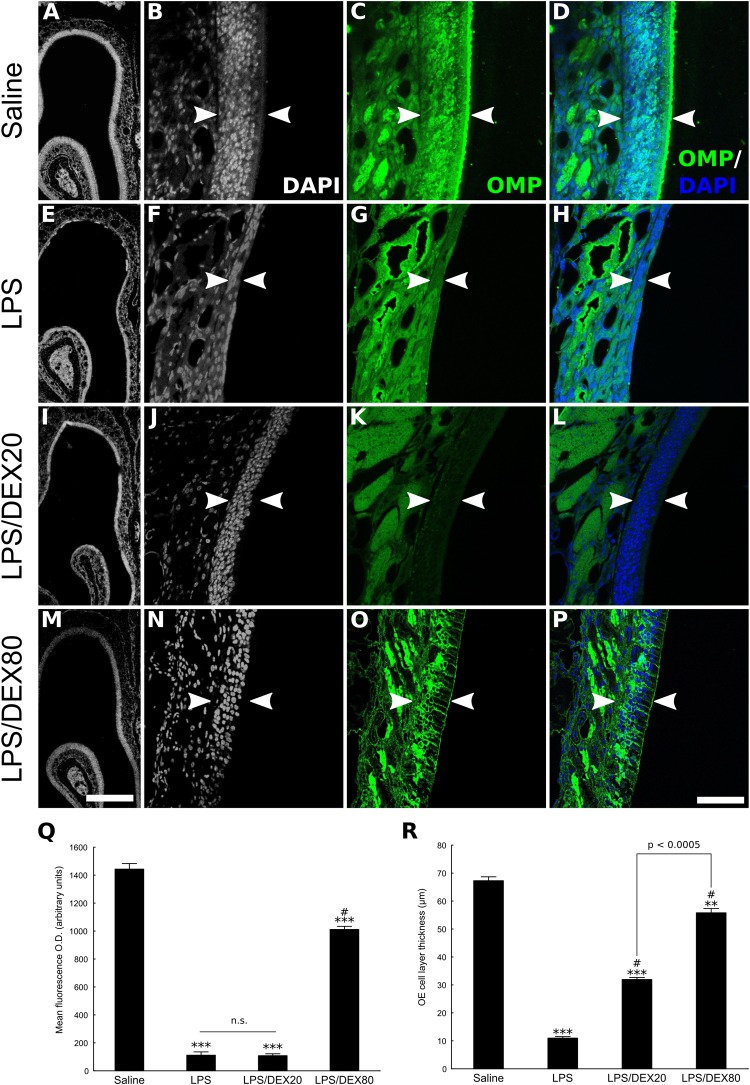
Topical DEX reduces OE injury provoked by LPS intranasal infusion. The panel depicts the OE of control or LPS-treated animals, as delimited by arrowheads **(A–P)**. **(A,E,I,M)** Low magnification photomicrographs of DAPI-stained sections, revealing significant OE atrophy due to cell loss caused by the TLR4 agonist. These histological changes correlate with loss of OMP staining **(E–H)**. DEX, co-infused with LPS at the concentration of 20 or 80 ng/μl, as indicated, dose-dependently prevented the cell loss associated with the inflammatory lesion **(I–P)**. **(D,H,L,P)** Merged images of DAPI nuclear staining and OMP labeling. It should be noted that **A**, **E**, **I**, and **M** are not necessarily matched to high magnification photomicrographs. **(Q)** Mean optical density from OE sections labeled with antibody against the neuronal marker OMP. **(R)** OE cell layer thickness measures. Results represent means ± SEM of three mice per group. One-way ANOVA [*F*(3,8) = 627, *p* < 0.0001 for OD; *F*(3,8) = 509, *p* < 0.0001 for OE thickness] followed by a Tukey’s HSD multiple comparison test: significantly different (^∗∗^*p* < 0.001, ^∗∗∗^*p* < 0.0005) from the saline-treated animals; significantly different (^#^ *p* < 0.0005) from the LPS-treated group. Scale bars: 300 μm for low magnification pictures and 50 μm for all the others.

In order to evaluate topical DEX effects as anti-inflammatory drug, we first developed a fast and reversible inflammatory lesion model. Acute nasal infusion of 100 ng of LPS was able to produce extensive OSNs loss 48 h after treatment, mainly at the ipsilateral site of infusion, while saline infused OE remained intact (**Figure 1** and Supplementary Figure S1). OMP staining failed to detect mature OSNs in extensive areas of the OE from the LPS-treated group. In addition, DAPI nuclear staining revealed remarkable atrophy of the epithelium. Murine OE recovered rapidly after lesion, since OSNs were considerably regenerated 14 days after the infusion (Supplementary Figure S1). We must emphasize, however, that depending on the conditions (e.g., different LPS batches), the lesions were only reproducible at much higher doses (1–5 μg/nostril) according to our experience. The variability on LPS responses in nervous tissue has been recently discussed (Goldstein et al., 2017). For the study reported here, we were able to keep the same LPS dose (0.1 μg) for all *in vivo* protocols.

Dexamethasone co-treated animals showed dose-dependent protection against the inflammatory insult as evaluated by OMP staining and OE thickness measures (**Figures 1A–P**). Although the LPS/DEX 80 ng/μl group showed some spots of OMP-negative regions, the OE thickness remained largely preserved compared to the OE from animals that did not receive the drug. It is important to note that since DEX dose of 20 ng/μl only provides modest protection to the OE, as shown by OE thickness measures and lack of OMP preservation (**Figures 1Q,R**), our subsequent studies were conducted with an intermediate dose of 40 ng/μl. This would be compatible with a strategy of keeping DEX dosing as low as possible, ensuring the anti-inflammatory effect in our experimental conditions.

### Short-Term DEX Treatment After OE Injury Impairs OSN Regeneration

**FIGURE 2:**
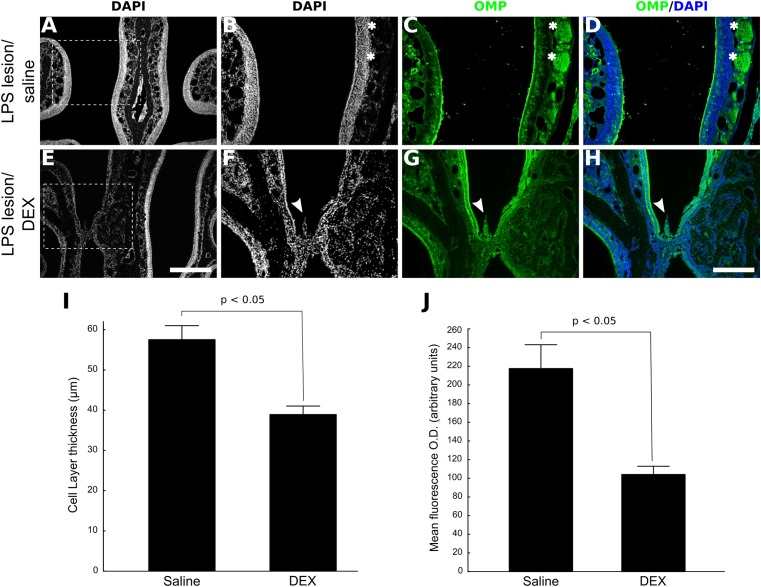
DEX treatment impairs OE regeneration after nasal injury mediated by LPS. Low magnification photomicrographs of DAPI-stained section revealing partial OE regeneration 10 days after LPS infusion **(A)**. Significant impairment of OSNs replenishment was observed when DEX (40 ng/μl) was infused three times, once a day, starting 48 h after LPS intranasal administration. **(C,G)** The photomicrographs represent main differences in OMP expression between the groups. It is also possible to note that DEX treatment promoted histological anomalies, but only in the LPS/DEX(3×)-treated group **(E–H)**. **(D,H)** Merged images of DAPI nuclear staining and OMP labeling. **(I)** OE cell layer thickness measures. **(J)** Mean optical density from OE sections labeled with the neuronal marker OMP. Results represent thickness means ± SEM of three mice per group. Statistical scores were obtained with Student’s *t*-test (*p* < 0.05 in **I** and **J**). Scale bars: 300 μm for low magnification pictures and 150 μm for all the others. Dashed squares indicate regions magnified for photomicrographs in **B–D** and **F–H**. White arrowheads indicate morphologically compromised region; white asterisks indicate nerve bundles.

Based on the histological analysis, lesions induced by LPS were considered consistently established 48 h post-infusion. Subsequently, we tested whether topical administration of DEX could alter the course of OMP-positive cells reappearance 10 days after LPS treatment by infusing the drug at Days 2, 3, and 4 after LPS treatment. The comparisons were conducted at this stage (i.e., 10 days after LPS infusion) because regeneration is only partial (Supplementary Figure S1) and in consequence, it is more flagrant to reveal treatment differences. As depicted in **Figure 2**, intranasal bolus infusion of DEX (40 ng/μl) repeated for another 2 consecutive days lead to histological changes of the partially regenerated OE. We could observe that tissue deformities, not found in the vehicle group, developed in every DEX-treated animal during lesion recovery, mainly at the level of the septum. Repeated DEX application after LPS lesion resulted in atrophic OE, and in some cases, cellular changes included the incursions from cells of the lamina propria. The atrophy also reflects marked absence of OMP-positive cells, implying a failure in efficient new OSNs generation in the injured region (**Figure 2**).

**FIGURE 3:**
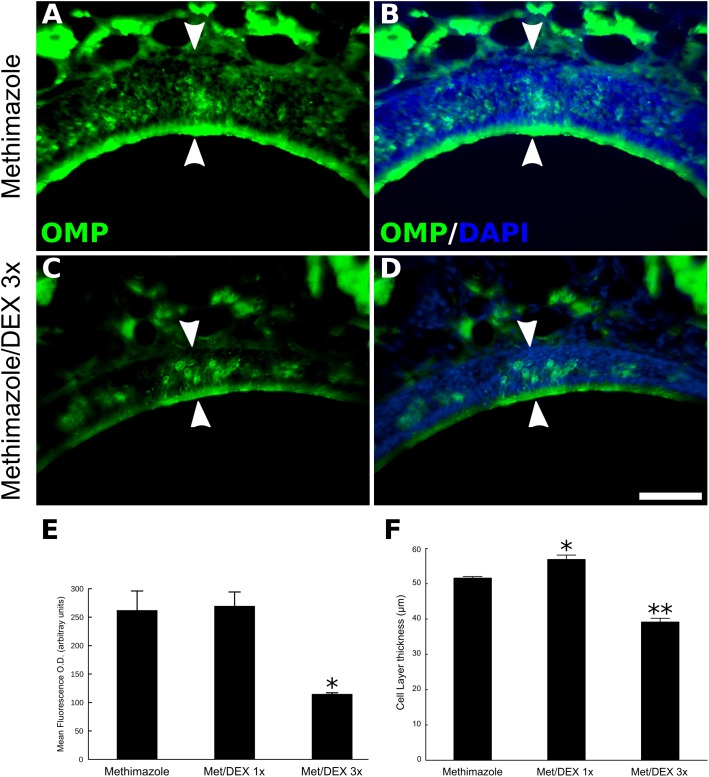
DEX short-term treatment, but not single-day treatment, impairs OE regeneration after OSNs loss induced by methimazole. Animals were treated with methimazole (50 mg/kg; two doses, 2 days a part) and sacrificed 14 days after the second intraperitoneal administration of the damaging compound. OMP-stained sections **(A–D)** from the OE reveal impaired cellular replenishment in topical DEX (40 ng/μl) 3 days-treated group **(C,D)** compared to the control animals **(A,B)**. **(B,D)** DNA staining with DAPI was merged to OMP in order to identify the OE cell layer. Mean optical density from OE sections labeled with the neuronal marker OMP **(E)** and OE cell layer thickness measurements **(F)** reveal that 1-day single DEX treatment, 24 h after the second injection of methimazole (1 dpl), increases OE thickness compared to the OE of mice that did not receive DEX. One-way ANOVA (*F*(2,6) = 13.2, *p* < 0.005 for OD; *F*(2,6) = 101, *p* < 0.0001 for OE thickness) followed by a Tukey’s HSD multiple comparison test: significantly different (^∗^*p* < 0.05, ^∗∗^*p* < 0.0005) from saline-/methimazole-treated animals. Scale bars: 50 μm. White arrowheads delimitate the OE.

Taking advantage of a drug-induced model of OE lesion (Brittebo, 1995), we also tested if DEX effects on neuronal regeneration are reproducible in general models or if they are specific for inflammatory lesions. Methimazole treatment causes virtually total loss of OMP-positive neurons in the OE (**Figure 3**). Two weeks after the drug treatment OSNs repopulate the OE, albeit not completely, making it possible to measure improvement or impairment of the regenerative process. Once again, topical DEX (40 ng/μl) short-term treatment reduced the overall reappearance of OMP-positive cells at the ipsilateral site of infusion (**Figures 3A–D**). Interestingly, 1-day single DEX infusion 24 h after the second injection of methimazole (1 dpl) is not able to reproduce the effect observed with 3 days treatment (**Figures 3E,F**). The animals from this group received saline solution infusion at 2 and 3 dpl. Surprisingly, we observed a trend for improvement in case of 1-day single treatment, since the OE thickness from first-day-only DEX group was slightly larger than methimazole group (**Figure 3F**).

### DEX Treatment Interferes With OE Neurogenesis Triggered by Injury

**FIGURE 4:**
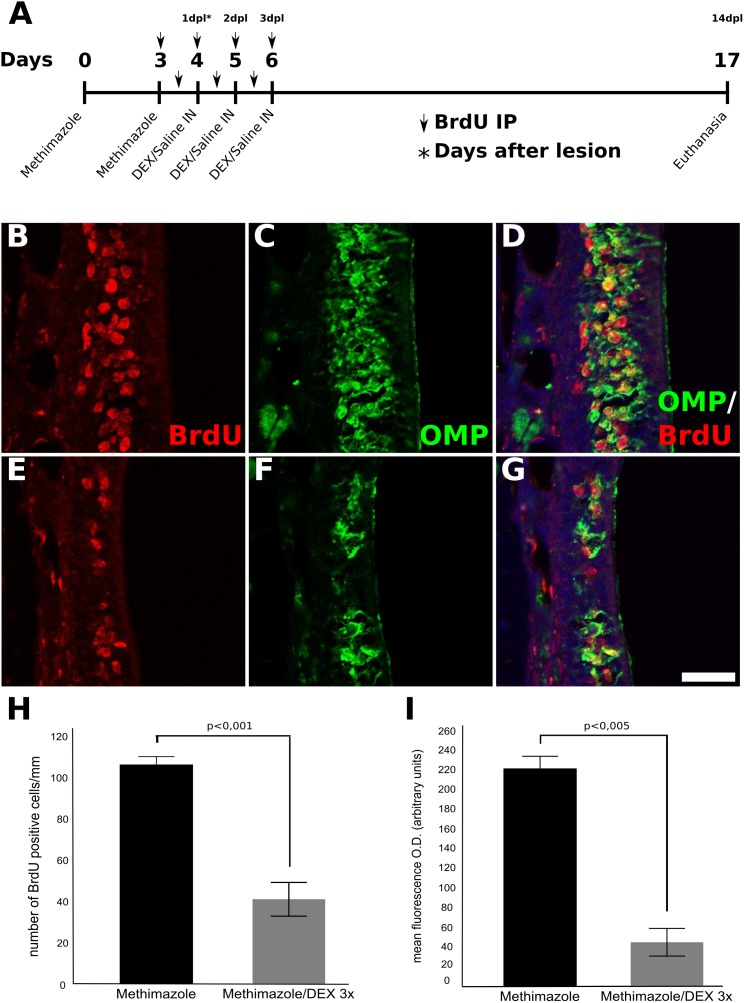
Fate-mapping of OE progenitor cells labeled with BrdU during lesion establishment and DEX treatment. Mice were treated with methimazole (two doses, 2 days a part) in order to provoke OSNs cell death, and euthanized 14 days after the second administration of methimazole (i.e., 14 dpl). The animals received BrdU i.p. injections twice a day as represented over the time-scale **(A)**. Animals that received intranasal DEX 40 ng/ml per day at 1, 2, and 3 dpl showed a pronounced decrease number of BrdU-stained cells compared to their controls **(B–G)**. BrdU-labeled cells were found to express OMP in a significant fraction of cells **(D,G)** supporting decreased neurogenesis from precursor cells treated with DEX and revealing that the corticoid did not change the neuronal cell fate. **(H)** Quantification of the number of BrdU-positive cells in the OE, **(I)** mean O.D. of OMP staining. Results represent thickness means ± SEM of three mice per group. Statistical scores were obtained with Student’s *t*-test. Scale bar: 50 μm.

In order to perform fate mapping of cells generated in the course of DEX treatment, the thymidine analog BrdU was incorporated into dividing cells during methimazole lesion establishment and DEX treatment. This strategy labels dividing progenitor cells recruited by the OE injury stimulus, and in consequence, allows the evaluation of DEX effects on cell proliferation, survival, or differentiation. As shown in **Figure 4**, a smaller number of BrdU-positive cells was observed in DEX-treated OE (**Figures 4E–G**) when compared to non-treated control tissue (**Figures 4B–D**) 14 days after the second methimazole infusion (i.e., 14 dpl). We further assessed BrdU-/OMP-double-labeling in order to verify if DEX treatment could impair neuronal progenitor differentiation or dramatically change cellular fate. We observed predominance of OMP-/BrdU-double-labeled OSNs in both DEX-treated and control animals (**Figures 4D,G**), indicating that labeled progenitor cells differentiated into mature OSNs.

### DEX Treatment Interferes With Progenitor Cell Proliferation Without Inducing TUNEL-Positive Cells

**FIGURE 5:**
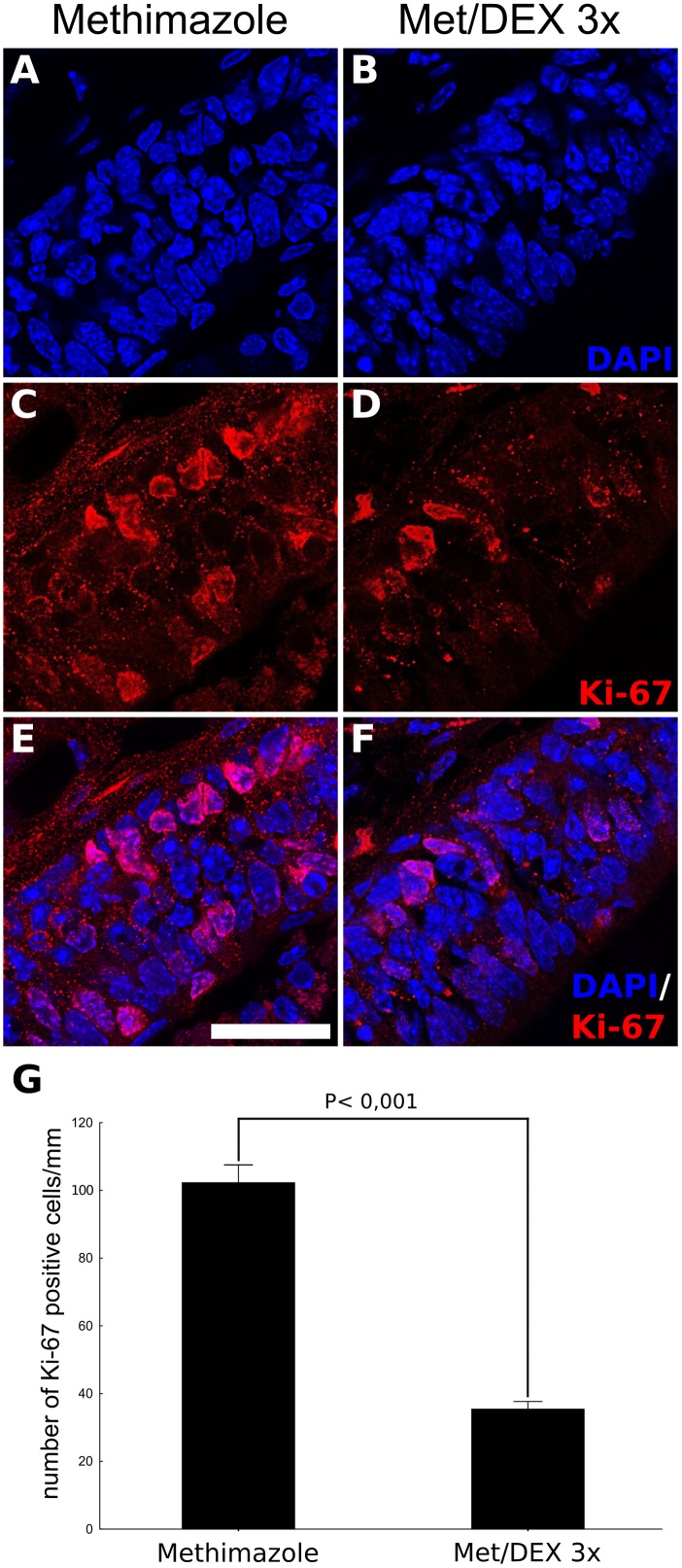
DEX promotes reduced OE progenitor cell-cycling activity triggered by injury. DEX or saline were infused in mice nostrils after methimazole lesion, according to a 3-day repeated treatment, once a day, starting from 1 dpl. The animals were subjected to euthanasia 6 h after the third intranasal infusion. DAPI nuclear staining combined to Ki-67 immunostaining, used here as an index of cell division and proliferative activity, showed reduced number of positive cells lining the OE lamina basal in case of DEX treatment **(A–F)**. The cell counting was adjusted to linear extension of OE-selected regions **(G)**. Results represent means ± SEM. Statistical scores were obtained with Student’s *t*-test. Scale bar: 15 μm.

**FIGURE 6:**
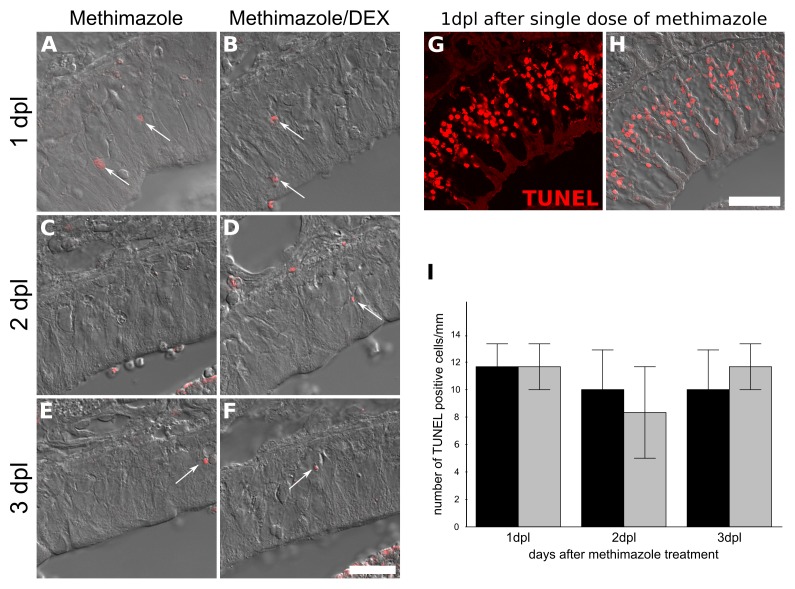
Topical DEX is not associated with increase in TUNEL-positive cells in injured OE. TUNEL labeling for DNA breaks was used as an index of cell death. Methimazole-lesioned OE did not show increase in TUNEL-positive cells upon the GC treatment (ANOVA *F*(1,12) < 1 × 10^-6^, *p* = 0.9994). We failed to detect changes at 1, 2, or 3 dpl **(A–F,I)**. In order to validate the technic, we used 24 h methimazole single bolus administration as positive control **(G,H)**, which showed several positive nuclei. The cell counting was adjusted to linear extension of OE-selected regions **(I)**. Results represent means ± SEM. Scale bar: 20 μm. White arrows show few TUNEL-positive nuclei.

Since BrdU labeling revealed a dramatic decrease in the number of OSNs derived from mitogenic activity during lesion establishment and intranasal treatment, we next evaluated if DEX interfered with cell division. The number of OE Ki-67^+^ cells associated to basal lamina, the region populated by HBCs and GBCs progenitors, were counted in order to reveal active cell division associated with OSN replenishment (**Figure 5**). The corticoid provoked a decrease in the number of Ki-67^+^ cells as evaluated at the 3 dpl (6 h after the third DEX administration), indicating reduced cell-cycling activity. In contrast, the analysis of TUNEL-positive cells, a marker of DNA breaks suggestive of apoptosis and other types of cell death, failed to reveal DEX effects on cell death (**Figure 6**).

### Protein Synthesis and S6-Kinase Are Down-Regulated by DEX at Specific Time-Points

**FIGURE 7:**
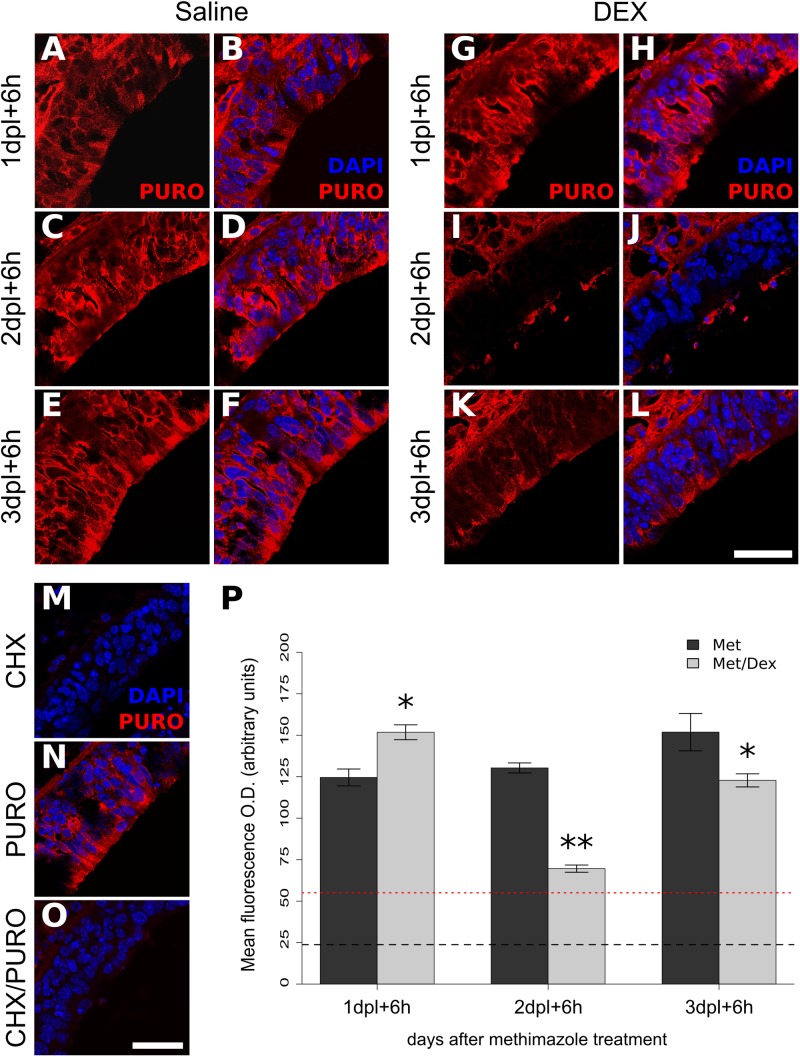
Protein synthesis is modulated by DEX treatment in time-dependent manner. Puromycin tagging was employed *in vivo* in order to track translation activity *in situ*. Mice received intranasal treatment of DEX or saline from 1 to 3 dpl and were subjected to puromycin (PURO) intranasal infusion 6 h after the DEX or saline topical treatment at time-points 1, 2, and 3 dpl. The animals were euthanized 30 min thereafter and the tissue processed for immunofluorescence with anti-puromycin antibody **(A–L)**. Signal specificity was verified with CHX pre-treatment **(M–O)**. Mean O.D. from OE sections labeled with antibody against PURO **(P)** shows dramatic decrease in translation at 2 dpl. Results represent means ± SEM (*n* = 3). Dashed line represents mean O.D. of background staining in the absence of PURO infusion. Red-dotted line represents mean O.D. of PURO incorporation in the presence of CHX. Two-way ANOVA [*F*(2,12) = 461, *p* < 0.0001 for interaction between treatment and time] followed by a Tukey’s HSD multiple comparison test: significantly different (^∗^*p* < 0.05, ^∗∗^*p* < 0.001) from methimazole-treated animals. Scale bars: 20 μm.

**FIGURE 8:**
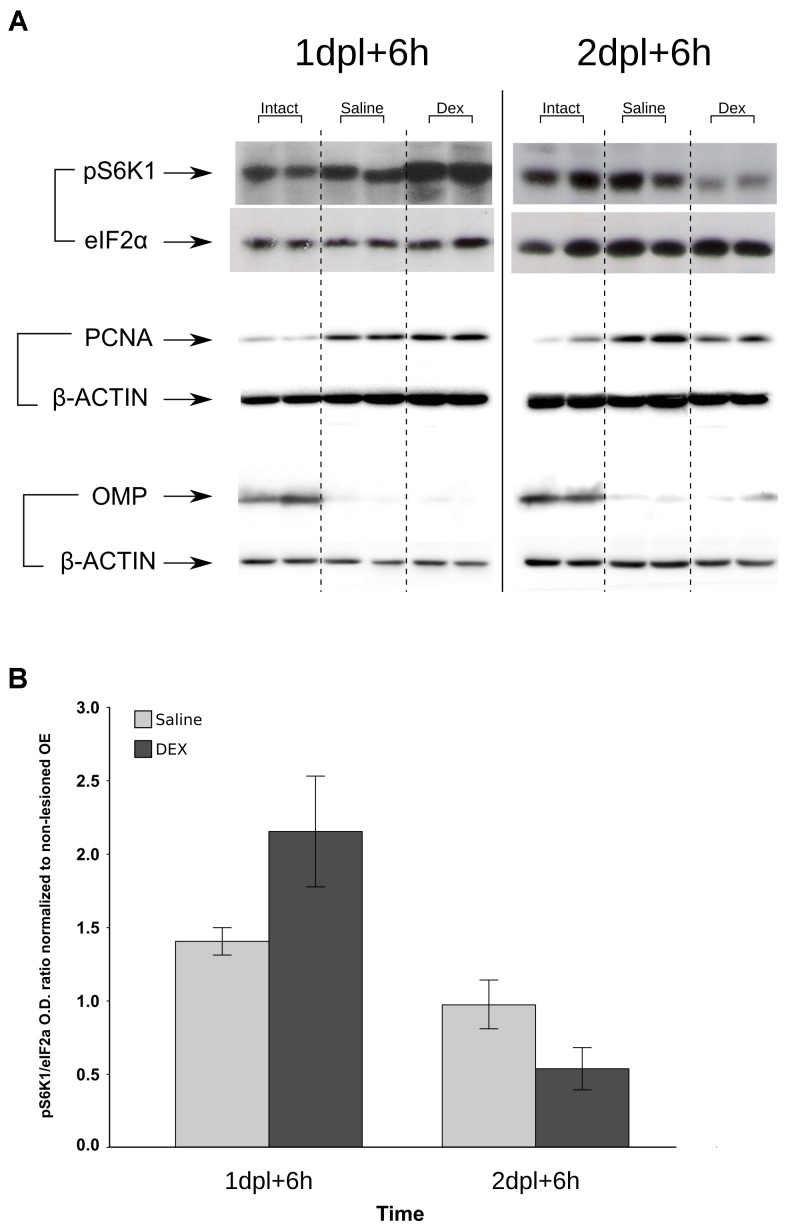
Deregulation of S6K1 phosphorylation levels by DEX treatment to the injured OE. Samples from methimazole-treated mice that received saline or DEX intranasal infusion at 1 and 2 dpl were resolved in SDS–PAGE. The proteins were transferred to PVDF membranes, which were probed for phospho-S6K1 or eIF2α as loading controls **(A)**. Densitometric analysis of pS6K1 levels corrected for eIF2α and normalized against intact OE controls **(B)** shows signal decrease at 2 dpl, similar to that of protein synthesis (**Figure 7**). PCNA was used as an index of cell proliferation and loss of OMP signal as control for lesion establishment (for each immunoblot β-actin was used as loading control). Images are representative of *n* = 3 (each independent sample was a pool from two mice). Image crop edition was performed to enable lane grouping according to treatments (each representative result showing six bands and the loading controls are from the same membrane). Graphic results represent means ± SEM. Statistical analysis revealed a significant interaction between the factors treatment (saline or DEX) and time (1 or 2 dpl); *F*(1,8) = 6.888, *p* = 0.030.

The protein synthesis pathway is a key biological process associated with cell proliferation. We connected the negative effects of DEX treatment with protein synthesis using an *in situ* approach to label nascent proteins/peptides with puromycin (Starck et al., 2004). A similar strategy has showed strong staining in cells with highly proliferative rates in intestine (Liu et al., 2012). Puromycin labeling revealed a robust decrease in translation following the second DEX infusion (**Figures 7A–L,P**). Signal specificity was confirmed by the poor labeling when the nostrils were treated with protein synthesis inhibitor CHX (**Figures 7M–O**). A single DEX infusion is not able to promote the same effect and showed the opposite trend, while the effect magnitude of the third DEX infusion was milder than the observed the second day (**Figure 7P**). In order to corroborate the specific shutdown in translation at the second, but not at the first day of DEX treatment, we verified the levels of phosphorylated p70-S6 kinase (pS6K1), a downstream effector molecule of the mTOR complex 1 (mTORC1) pathway, which regulates protein synthesis and cell proliferation. As depicted in **Figure 8**, S6K1 followed contrasting regulations comparing the first and second consecutive day of DEX treatment. The decrease in pS6K1 levels was only observed at the time-point corresponding to the second DEX infusion. It is worth noting the coincident increase or decrease in the PCNA levels, another marker of cell division, according to treatment and time-point that influenced the levels of S6K1 phosphorylation (**Figure 8**). In addition, we also observed that repeated intranasal infusion of rapamycin, an inhibitor of mTORC1, was detrimental to OSNs recovery following methimazole-induced lesion (Supplementary Figure S2).

### *In Vitro* DEX Exposure Dose-Dependently Interferes With OE Neuronal Progenitor Activity Through GR

**FIGURE 9:**
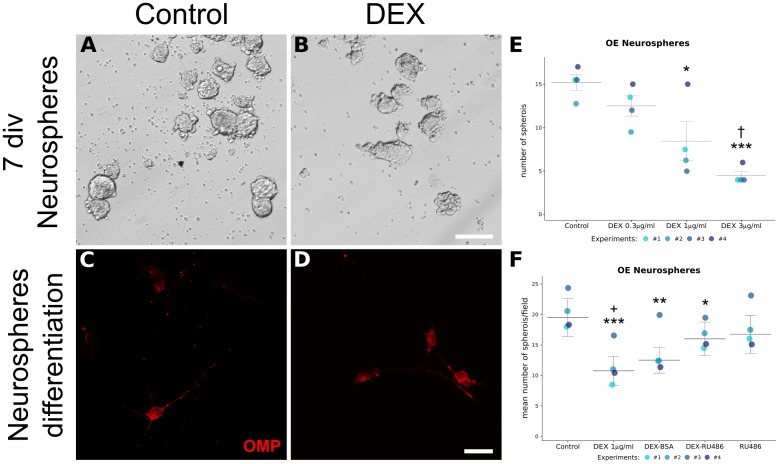
*In vitro* generation of OE neurospheres recapitulates GR-mediated decrease in progenitor cell proliferation. Nasal mucosa from newborn mice were pooled for each independent experiment, dissociated, and split in culture plates according to the indicated treatments. **(A,B)** Representative photomicrographs of OE spheroids culture in the presence of growth factors and DEX, or its vehicle, for 7 div. **(C,D)** OMP immunostaining of differentiated cells derived from neurospheres. OMP-positive cells were observed in both treatments. Spheroids counting were plotted for independent experiments as shown in **E** and **F**. DEX dose-dependently reduced the number of OE neurospheres **(E)**, an effect prevented by pre-incubation with GR antagonist RU486 **(F)**. Data are represented as median ± IQR. Statistical analysis was performed as replicated blocks (Quade test: *F*(3,9) = 14.5, *p* = 0.0008663 for data in **E**; *F*(4,12) = 14.6, *p* = 0.0001473 for data in **F**) and comparison significance was adjusted with Holm method (^∗^*p* < 0.05, ^∗∗^*p* < 0.005, ^∗∗∗^*p* < 0.001 compared to control; ^†^*p* < 0.05 compared to DEX 0.3 μg/ml; ^+^*p* < 0.05 compared to DEX-RU486). Scale bar: **(A,B)** 100 μm; **(C,D)** 25 μm.

Since *in vivo* experiments cannot rule out indirect DEX effects, even when infused locally, we performed *in vitro* assays using neurosphere formation as a correlative index of progenitor cell proliferation. Another advantage of this approach is to dissociate GC effects from its anti-inflammatory activity. In essence, immune cells are not representative cell types in this preparation. We incubated the cell suspension with DEX at varied standard concentrations and verified a dose-dependent decrease in spheroid counting (**Figures 9A,B,E**). These neurospheres could generate OMP-positive cells, independently of the treatment (**Figures 9C,D**). We also verified if DEX mediated its effects through GR using the antagonist mifepristone (RU486). In attempt to verify if membrane-associated GR was involved, DEX-BSA was also used. RU486 was able to prevent, albeit not completely, DEX effects on neurosphere formation (**Figure 9F**). While DEX-BSA presented similar effects to DEX, we cannot rule out that the complex is endocytosed with subsequent DEX release, especially because the incubation time was long (up to 7 div; please refer to the section “Discussion”).

## Discussion

Here, we developed a fast inflammatory model of OE lesion, which resembles the natural response to infections or acute inflammatory insults to the nasal mucosa. The ability of this bacterial toxin to cause extensive neuronal damage is in contrast to the fact that even higher LPS doses are in general not harmful to neuronal cells in the CNS (Szczepanik et al., 1996; Glezer et al., 2003b; Nadeau and Rivest, 2003). Very recently, another study showed that repeated LPS intranasal infusion (μg range) promoted rhinitis that damaged synaptic connection to the olfactory bulb (Hasegawa-Ishii et al., 2017), which is in agreement with the lesion results reported in this study.

As expected, DEX prevented LPS effects due to its notorious anti-inflammatory effects (Pang et al., 2012), including its previously shown ability to downregulate NF-κB activation in the brain (Glezer et al., 2003a). Although a detailed dose–response curve would be required in order to determine the minimal DEX effective dose, it is reasonable to suppose that the higher dose (80 ng/μl; ∼155 μM of sodium DEX–phosphate) used in our study is compatible with a therapeutic range, since the lower dose (20 ng/μl) failed to prevent inflammatory-induced loss of OMP staining. In addition, DEX intranasal aqueous spray preparation designed to deliver 800 μg/day in humans (Martino et al., 2015), as well DEX commercial ophthalmic preparations, contains 1 μg/μl of the drug, which is more than 10 times concentrated than the higher dose used in our study. Although 80 ng/μl would be therapeutically relevant, we selected an intermediate dose of 40 ng/μl to assess DEX effects in the course of neuronal regeneration.

The OE is quite efficiently repaired 14 days after the inflammatory stimulus, which is in agreement with the fast neuronal regeneration observed in a transgenic model of reversible chronic rhinosinusitis (Lane et al., 2010). Hence, we analyzed the time-point of 10 days post-infusion (LPS) to better access different degrees of OSN regeneration. Indeed, striking differences were observed when DEX was infused 2, 3, and 4 days after LPS infusion. It is possible that this period is critical for organizing regenerative responses that are in some way compromised by DEX treatment. Interestingly, a recent study showed that inflammation-driven NF-κB activity is necessary for OE regeneration (Chen et al., 2017). Therefore, we investigated whether the drug effect is mediated through its anti-inflammatory effects or other mechanisms, GR-dependent or not.

Methimazole has been widely used to induce reproducible OSN loss and offers a straightforward paradigm to test the DEX effects (Murray and Calof, 1999; Leung et al., 2007; Chen et al., 2017). In this case, DEX effects could be analyzed by infusing the drug when the lesion was definitively and uniformly established throughout the entire OE. It is important to note that the OE takes a longer time to regenerate after methimazole administration when compared to LPS infusion. Despite this dissimilarity and the different nature of the damages, DEX effects on neuronal regeneration were comparable between the lesion models. This implies that specific cellular dynamics involved particularly in LPS or inflammatory lesion models does not account solely for the observed effects of the steroid. It is therefore more likely that the presence of the drug in a specific period, shortly after the lesion, is the underlying cause that hampers repair mechanisms in the OE. The fast organization of both proliferative and fate determining transcriptional changes has been previously reported during OE recovery after bulbectomy (Shetty et al., 2005), and closely resembles the period in which DEX was infused. Our data also suggest that topic DEX administration must be recurrent during this crucial stage in order to interfere with OSNs replenishment. However, it remains to be determined whether repeated administrations or DEX topic delivery at specific time-points after lesion explains the dramatic effects of the steroid on OE regeneration.

Fate analysis of cells derived from BrdU-labeled progenitors revealed a reduced number of labeled cells in the OE after a short-term DEX 3-days treatment. Our results indicate that DEX short-term treatment does not halt the differentiation process, since both fate-mapping and *in vitro* results indicate that DEX-treated progenitor cells can differentiate into OSNs. The reduced number of regenerated OSNs can be a net result of diminished proliferation of progenitor cells or increased cell death. One study showed that GCs promote apoptosis in the OE (Dorscheid et al., 2003), while in another study this effect was curiously associated with a better regeneration of the OE (Takanosawa et al., 2009). Our results point to a different effect, which we believe may reflect the accuracy of our model. Reduction of Ki-67^+^ cells after DEX 3-days treatment and failure in detecting increased TUNEL staining during the 3 days of steroid treatment strongly suggests that GCs main effect is on progenitor cell proliferation. We also corroborated the effect *in vitro*, excluding DEX anti-inflammatory mechanisms from our observations. We further explored associated cellular changes and found a profound reduction in protein synthesis caused by the second DEX infusion at 2 dpl. A remarkable correlation was found with pS6K1 levels, a mTORC1 downstream effector molecule involved in protein synthesis through S6 ribosomal protein phosphorylation and cell proliferation (Saxton and Sabatini, 2017). Interestingly, genetically driven activation of mTOR pathway favors axonal regeneration in a model of optic nerve injury (Park et al., 2008). Here, we propose that mTOR pathway activation could be explored for OE function preservation, since DEX modulates mTORC1 targets (**Figures 7**, **8**) and rapamycin interferes with OSNs replenishment after methimazole lesion (Supplementary Figure S2).

Delay in olfactory function recovery is a risk factor for olfactory loss (London et al., 2008). Even more, DEX and GCs have been shown to interfere with neurogenesis in other models (Kim et al., 2004; Schroter et al., 2009; Yu et al., 2010), which is in agreement with our findings. Interestingly, a clinical study found remarkable differences in the outcomes of topical steroid-treated patients versus those receiving systemic treatment. It has also been reported that systemic GCs are beneficial to OE regeneration or olfactory nerve recovery (Carbone et al., 2003; Heilmann et al., 2004; Kapucu et al., 2012). The route of administration seems to be very important for reproducing DEX effects, which appears to be hazardous to neuronal recovery independently of the OE lesion model. Here, we also explored DEX effects *in vitro*, which supports cell-autonomous mechanisms. The effects were GR-dependent since the antagonist largely prevented reduction in progenitor activity. While a membrane-associated GR has been implicated in DEX non-genomic effects on neural precursor cells (Samarasinghe et al., 2011), we cannot assure that DEX-BSA was not processed and released the GC. This possibility remains to be verified in future studies.

## Conclusion

Our study supports that the safety of topical steroidal anti-inflammatory drugs must be further evaluated in terms of olfactory function recovery. DEX, selected here as a representative drug to explore its impact on regenerative outcomes, is able to abrogate important repair mechanisms of the OE. Although GCs are important for controlling the inflammatory component of rhinosinusitis, a risk-benefit analysis must account the systemic side-effects of this class of drug and the possibility of anosmia and hyposmia development over recurrent treatments.

## Author Contributions

UC, AX, FdS, TC, and SC performed the experiments. UC, AX, MP, BC, BM, and IG designed the experiments. UC and AX prepared the figure panels. UC, AX, MP, BC, BM, and IG analyzed the data. UC, BM, and IG wrote the manuscript.

## Conflict of Interest Statement

The authors declare that the research was conducted in the absence of any commercial or financial relationships that could be construed as a potential conflict of interest.

## Footnotes

1 http://rsbweb.nih.gov/ij/

2 http://www.gimp.org/

3 http://inkscape.org

4 http://rsbweb.nih.gov/ij/

5 http://fiji.sc/Fiji

6 https://www.gnu.org/software/pspp

## Abbreviations

CHX: cycloheximide
CNS: central nervous system
DEX: dexamethasone
DEX-BSA: dexamethasone conjugated to bovine serum albumin
Div: days *in vitro*
Dpl: days post-lesion
GC: glucocorticoid
GR: glucocorticoid receptor
LPS: lipopolisaccharide
OE: olfactory epithelium
OMP: olfactory marker protein
OSN: olfactory sensory neuron
PCNA: proliferating cell nuclear antigen
S6K1: p70-S6 ribosomal kinase
TLR: *Toll*-like receptor.

## References

[B1] Aderem A. Ulevitch R. J. (2000). Toll-like receptors in the induction of the innate immune response. *Nature* 406 782–787. 10.1038/35021228 10963608

[B2] Almawi W. Y. Beyhum H. N. Rahme A. A. Rieder M. J. (1996). Regulation of cytokine and cytokine receptor expression by glucocorticoids. *J. Leukoc. Biol.* 60 563–572. 10.1002/jlb.60.5.563 8929546

[B3] Avery C. M. (1998). A controlled trial of antepartum glucocorticoid treatment for prevention of the respiratory distress syndrome in premature infants, by G. C. Liggins, MB, PhD, FRCOG, and R. N. Howie, MB, MRACP, Pediatrics, 1972;50:515-525. *Pediatrics* 102 250–251. 9651447

[B4] Bachert C. Pawankar R. Zhang L. Bunnag C. Fokkens W. J. Hamilos D. L. (2014). ICON: chronic rhinosinusitis. *World Allergy Organ. J.* 7 1–28. 10.1186/1939-4551-7-25 25379119 PMC4213581

[B5] Bergman U. Östergren A. Gustafson A. L. Brittebo E. (2002). Differential effects of olfactory toxicants on olfactory regeneration. *Arch. Toxicol.* 76 104–112. 10.1007/s00204-002-0321-2 11914780

[B6] Blomqvist E. H. Bramerson A. Stjarne P. Nordin S. (2004). Consequences of olfactory loss and adopted coping strategies. *Rhinology* 42 189–194. 15626250

[B7] Brann J. H. Firestein S. J. (2014). A lifetime of neurogenesis in the olfactory system. *Front. Neurosci.* 8:182 10.3389/fnins.2014.00182 PMC4071289 25018692

[B8] Brittebo E. B. (1995). Metabolism-dependent toxicity of methimazole in the olfactory nasal mucosa. *Pharmacol. Toxicol.* 76 76–79. 10.1111/j.1600-0773.1995.tb00107.x 7753763

[B9] Carbone M. Goss E. Carrozzo M. Castellano S. Conrotto D. Broccoletti R. (2003). Systemic and topical corticosteroid treatment of oral lichen planus: a comparative study with long-term follow-up. *J. Oral Pathol. Med.* 32 323–329. 10.1034/j.1600-0714.2003.00173.x 12787038

[B10] Chen M. Reed R. R. Lane A. P. (2017). Acute inflammation regulates neuroregeneration through the NF-kappaB pathway in olfactory epithelium. *Proc. Natl. Acad. Sci. U.S.A.* 114 8089–8094. 10.1073/pnas.1620664114 28696292 PMC5544262

[B11] Croy I. Nordin S. Hummel T. (2014). Olfactory disorders and quality of life-an updated review. *Chem. Senses* 39 185–194. 10.1093/chemse/bjt072 24429163

[B12] Csomor P. Sziklai I. Karosi T. (2013). Effects of intranasal steroid treatment on the presence of biofilms in non-allergic patients with chronic rhinosinusitis with nasal polyposis. *Eur. Arch. Otorhinolaryngol.* 271 1057–1065. 10.1007/s00405-013-2666-y 23978952

[B13] De Bosscher K. Vanden Berghe W. Haegeman G. (2000). Mechanisms of anti-inflammatory action and of immunosuppression by glucocorticoids: negative interference of activated glucocorticoid receptor with transcription factors. *J. Neuroimmunol.* 109 16–22. 10.1016/S0165-5728(00)00297-6 10969176

[B14] De Bosscher K. Vanden Berghe W. Haegeman G. (2003). The interplay between the glucocorticoid receptor and nuclear factor-kappaB or activator protein-1: molecular mechanisms for gene repression. *Endocr. Rev.* 24 488–522. 10.1210/er.2002-0006 12920152

[B15] Dorscheid D. R. Low E. Conforti A. Shifrin S. Sperling A. I. White S. R. (2003). Corticosteroid-induced apoptosis in mouse airway epithelium: effect in normal airways and after allergen-induced airway inflammation. *J. Allergy Clin. Immunol.* 111 360–366. 10.1067/mai.2003.117 12589357

[B16] Ekdahl C. T. Kokaia Z. Lindvall O. (2009). Brain inflammation and adult neurogenesis: the dual role of microglia. *Neuroscience* 158 1021–1029. 10.1016/j.neuroscience.2008.06.052 18662748

[B17] Fandino M. Macdonald K. I. Lee J. Witterick I. J. (2013). The use of postoperative topical corticosteroids in chronic rhinosinusitis with nasal polyps: a systematic review and meta-analysis. *Am. J. Rhinol. Allergy* 27 146–157. 10.2500/ajra.2013.27.3950 24119596 PMC3899544

[B18] Franklin R. J. Ffrench-Constant C. (2008). Remyelination in the CNS: from biology to therapy. *Nat. Rev. Neurosci.* 9 839–855. 10.1038/nrn2480 18931697

[B19] Glezer I. Lapointe A. Rivest S. (2006). Innate immunity triggers oligodendrocyte progenitor reactivity and confines damages to brain injuries. *FASEB J.* 20 750–752. 10.1096/fj.05-5234fje 16464958

[B20] Glezer I. Munhoz C. D. Kawamoto E. M. Marcourakis T. Avellar M. C. Scavone C. (2003a). MK-801 and 7-Ni attenuate the activation of brain NF-kappa B induced by LPS. *Neuropharmacology* 45 1120–1129. 14614955 10.1016/s0028-3908(03)00279-x

[B21] Glezer I. Zekki H. Scavone C. Rivest S. (2003b). Modulation of the innate immune response by NMDA receptors has neuropathological consequences. *J. Neurosci.* 23 11094–11103. 14657167 10.1523/JNEUROSCI.23-35-11094.2003 PMC6741035

[B22] Goldstein E. Z. Church J. S. Pukos N. Gottipati M. K. Popovich P. G. McTigue D. M. (2017). Intraspinal TLR4 activation promotes iron storage but does not protect neurons or oligodendrocytes from progressive iron-mediated damage. *Exp. Neurol.* 298 42–56. 10.1016/j.expneurol.2017.08.015 28851597 PMC5658243

[B23] Graziadei P. P. C. Monti-Graziadei G. A. (1979). Neurogenesis and neuron regeneration in the olfactory system of mammals. I. Morphological aspects of differentiation and structural organization of the olfactory sensory neurons. *J. Neurocytol.* 8 1–18. 10.1007/BF01206454 438867

[B24] Hasegawa-Ishii S. Shimada A. Imamura F. (2017). Lipopolysaccharide-initiated persistent rhinitis causes gliosis and synaptic loss in the olfactory bulb. *Sci. Rep.* 7:11605. 10.1038/s41598-017-10229-w 28912588 PMC5599676

[B25] Heilmann S. Huettenbrink K.-B. Hummel T. (2004). Local and systemic administration of corticosteroids in the treatment of olfactory loss. *Am. J. Rhinol.* 18 29–33. 15035568

[B26] Holbrook E. H. Leopold D. A. (2006). An updated review of clinical olfaction. *Curr. Opin. Otolaryngol. Head Neck Surg.* 14 23–28. 10.1097/01.moo.0000193174.77321.39 16467634

[B27] Ihara S. Yoshikawa K. Touhara K. (2013). Chemosensory signals and their receptors in the olfactory neural system. *Neuroscience* 254 45–60. 10.1016/j.neuroscience.2013.08.063 24045101

[B28] Iwai N. Zhou Z. Roop D. R. Behringer R. R. (2008). Horizontal basal cells are multipotent progenitors in normal and injured adult olfactory epithelium. *Stem Cells* 26 1298–1306. 10.1634/stemcells.2007-0891 18308944 PMC4091843

[B29] Kapucu B. Cekin E. Erkul B. E. Cincik H. Gungor A. Berber U. (2012). The effects of systemic, topical, and intralesional steroid treatments on apoptosis level of nasal polyps. *Otolaryngol. Head Neck Surg.* 147 563–567. 10.1177/0194599812446678 22555894

[B30] Kim J. B. Ju J. Y. Kim J. H. Kim T.-Y. Yang B.-H. Lee Y.-S. (2004). Dexamethasone inhibits proliferation of adult hippocampal neurogenesis in vivo and in vitro. *Brain Res.* 1027 1–10. 10.1016/j.brainres.2004.07.093 15494151

[B31] Krolewski R. C. Jang W. Schwob J. E. (2011). The generation of olfactory epithelial neurospheres in vitro predicts engraftment capacity following transplantation in vivo. *Exp. Neurol.* 229 308–323. 10.1016/j.expneurol.2011.02.014 21376038 PMC3100381

[B32] Lane A. P. Turner J. May L. Reed R. (2010). A genetic model of chronic rhinosinusitis-associated olfactory inflammation reveals reversible functional impairment and dramatic neuroepithelial reorganization. *J. Neurosci.* 30 2324–2329. 10.1523/JNEUROSCI.4507-09.2010 20147558 PMC2957830

[B33] Leung C. T. Coulombe P. A. Reed R. R. (2007). Contribution of olfactory neural stem cells to tissue maintenance and regeneration. *Nat. Neurosci.* 6 720–726. 10.1038/nn1882 17468753

[B34] Liu J. Xu Y. Stoleru D. Salic A. (2012). Imaging protein synthesis in cells and tissues with an alkyne analog of puromycin. *Proc. Natl. Acad. Sci. U.S.A.* 109 413–418. 10.1073/pnas.1111561108 22160674 PMC3258597

[B35] London B. Nabet B. Fisher A. R. White B. Sammel M. D. Doty R. L. (2008). Predictors of prognosis in patients with olfactory disturbance. *Ann. Neurol.* 63 159–166. 10.1002/ana.21293 18058814

[B36] Mackay-Sim A. Kittel P. W. (1991). On the life span of olfactory receptor neurons. *Eur. J. Neurosci.* 3 209–215. 10.1111/j.1460-9568.1991.tb00081.x 12106197

[B37] Martino B. J. Church C. A. Seiberling K. A. (2015). Effect of intranasal dexamethasone on endogenous cortisol level and intraocular pressure. *Int. Forum Allergy Rhinol.* 5 605–609. 10.1002/alr.21514 25907564

[B38] Meltzer E. O. Charous B. L. Busse W. W. Zinreich S. J. Lorber R. R. Danzig M. R. (2000). Added relief in the treatment of acute recurrent sinusitis with adjunctive mometasone furoate nasal spray. *J. Allergy Clin. Immunol.* 106 630–637. 10.1067/mai.2000.109056 11031332

[B39] Monje M. L. (2003). Inflammatory blockade restores adult hippocampal neurogenesis. *Science* 302 1760–1765. 10.1126/science.1088417 14615545

[B40] Mori K. Sakano H. (2011). How is the olfactory map formed and interpreted in the mammalian brain? *Annu. Rev. Neurosci.* 34 467–499. 10.1146/annurev-neuro-112210-112917 21469960

[B41] Murray R. C. Calof A. L. (1999). Neuronal regeneration: lessons from the olfactory system. *Cell Dev. Biol.* 10 421–431. 10.1006/scdb.1999.0329 10497099

[B42] Nadeau S. Rivest S. (2003). Glucocorticoids play a fundamental role in protecting the brain during innate immune response. *J. Neurosci.* 23 5536–5544. 12843254 10.1523/JNEUROSCI.23-13-05536.2003 PMC6741270

[B43] Pang Y. Fan L. W. Zheng B. Campbell L. R. Cai Z. Rhodes P. G. (2012). Dexamethasone and betamethasone protect against lipopolysaccharide-induced brain damage in neonatal rats. *Pediatr. Res.* 71 552–558. 10.1038/pr.2012.9 22314662 PMC3609027

[B44] Parikh A. Scadding G. K. Darby Y. Baker R. C. (2001). Topical corticosteroids in chronic rhinosinusitis: a randomized, double-blind, placebo-controlled trial using fluticasone propionate aqueous nasal spray. *Rhinology* 39 75–79. 11486442

[B45] Park K. K. Liu K. Hu Y. Smith P. D. Wang C. Cai B. (2008). Promoting axon regeneration in the adult CNS by modulation of the PTEN/mTOR pathway. *Science* 322 963–966. 10.1126/science.1161566 18988856 PMC2652400

[B46] Pohlert T. (2014). *The Pairwise Multiple Comparison of Mean Ranks Package (PMCMR). R Package 27*. Available at: http://cran.ms.unimelb.edu.au/web/packages/PMCMR/vignettes/PMCMR.pdf

[B47] R Core Team (2017). *R: A Language and Environment for Statistical Computing*. Vienna: R Foundation Statistical Computing.

[B48] Rosewicz S. McDonald A. R. Maddux B. A. Goldfine I. D. Miesfeld R. L. Logsdon C. D. (1988). Mechanism of glucocorticoid receptor down-regulation by glucocorticoids. *J. Biol. Chem.* 263 2581–2584. 3343225

[B49] Russell R. C. Fang C. Guan K.-L. (2011). An emerging role for TOR signaling in mammalian tissue and stem cell physiology. *Development* 138 3343–3356. 10.1242/dev.058230 21791526 PMC3143559

[B50] Samarasinghe R. A. Di Maio R. Volonte D. Galbiati F. Lewis M. Romero G. (2011). Nongenomic glucocorticoid receptor action regulates gap junction intercellular communication and neural progenitor cell proliferation. *Proc. Natl. Acad. Sci. U.S.A.* 108 16657–16662. 10.1073/pnas.1102821108 21930911 PMC3189065

[B51] Saxton R. A. Sabatini D. M. (2017). mTOR signaling in growth, metabolism, and disease. *Cell* 168 960–976. 10.1016/j.cell.2017.02.004 28283069 PMC5394987

[B52] Scheinman R. I. Cogswell P. C. Lofquist A. K. Baldwin A. S. Jr. (1995). Role of transcriptional activation of I kappa B alpha in mediation of immunosuppression by glucocorticoids. *Science* 270 283–286. 10.1126/science.270.5234.283 7569975

[B53] Schroter A. Lustenberger R. M. Obermair F. J. Thallmair M. (2009). High-dose corticosteroids after spinal cord injury reduce neural progenitor cell proliferation. *Neuroscience* 161 753–763. 10.1016/j.neuroscience.2009.04.016 19364523

[B54] Shetty R. S. Bose S. C. Nickell M. D. McIntyre J. C. Hardin D. H. Harris A. M. (2005). Transcriptional changes during neuronal death and replacement in the olfactory epithelium. *Mol. Cell. Neurosci.* 30 583–600. 10.1016/j.mcn.2005.06.003 16456926

[B55] Shimizu N. Yoshikawa N. Ito N. Maruyama T. Suzuki Y. Takeda S. I. (2011). Crosstalk between glucocorticoid receptor and nutritional sensor mTOR in skeletal muscle. *Cell Metab.* 13 170–182. 10.1016/j.cmet.2011.01.001 21284984

[B56] Starck S. R. Green H. M. Alberola-Ila J. Roberts R. W. (2004). A general approach to detect protein expression in vivo using fluorescent puromycin conjugates. *Chem. Biol.* 11 999–1008. 10.1016/j.chembiol.2004.05.011 15271358

[B57] Szczepanik A. M. Fishkin R. J. Rush D. K. Wilmot C. A. (1996). Effects of chronic intrahippocampal infusion of lipopolysaccharide in the rat. *Neuroscience* 70 57–65. 10.1016/0306-4522(95)00296-U 8848136

[B58] Takanosawa M. Nishino H. Ohta Y. Ichimura K. (2009). Glucocorticoids enhance regeneration of murine olfactory epithelium. *Acta Otolaryngol.* 129 1002–1009. 10.1080/00016480802530663 19016360

[B59] Tsurufuji S. Sugio K. Takemasa F. (1979). The role of glucocorticoid receptor and gene expression in the anti-inflammatory action of dexamethasone. *Nature* 280 408–410. 10.1038/280408a0 460415

[B60] Wang H. Kubica N. Ellisen L. W. Jefferson L. S. Kimball S. R. (2006). Dexamethasone represses signaling through the mammalian target of rapamycin in muscle cells by enhancing expression of REDD1. *J. Biol. Chem.* 281 39128–39134. 10.1074/jbc.M610023200 17074751

[B61] Wickham H. (2009). *ggplot2 Elegant Graphics for Data Analysis*. New York, NY: Springer-Verlag 10.1007/978-0-387-98141-3

[B62] Xavier A. M. Anunciato A. K. O. Rosenstock T. R. Glezer I. (2016). Gene expression control by glucocorticoid receptors during innate immune responses. *Front. Endocrinol.* 7:31 10.3389/fendo.2016.00031 PMC4835445 27148162

[B63] Yu S. Patchev A. V. Wu Y. Lu J. Holsboer F. Zhang J. Z. (2010). Depletion of the neural precursor cell pool by glucocorticoids. *Ann. Neurol.* 67 21–30. 10.1002/ana.21812 20186952

