# Supplementary material for: Topical Dexamethasone Administration Impairs Protein Synthesis and Neuronal Regeneration in the Olfactory Epithelium
Source: Front Mol Neurosci. 2018 Mar 6;11:50. doi: 10.3389/fnmol.2018.00050 (PMC5845685; doi:10.3389/fnmol.2018.00050)
Supplement: Supplementary file 1 [file Image_1.pdf]

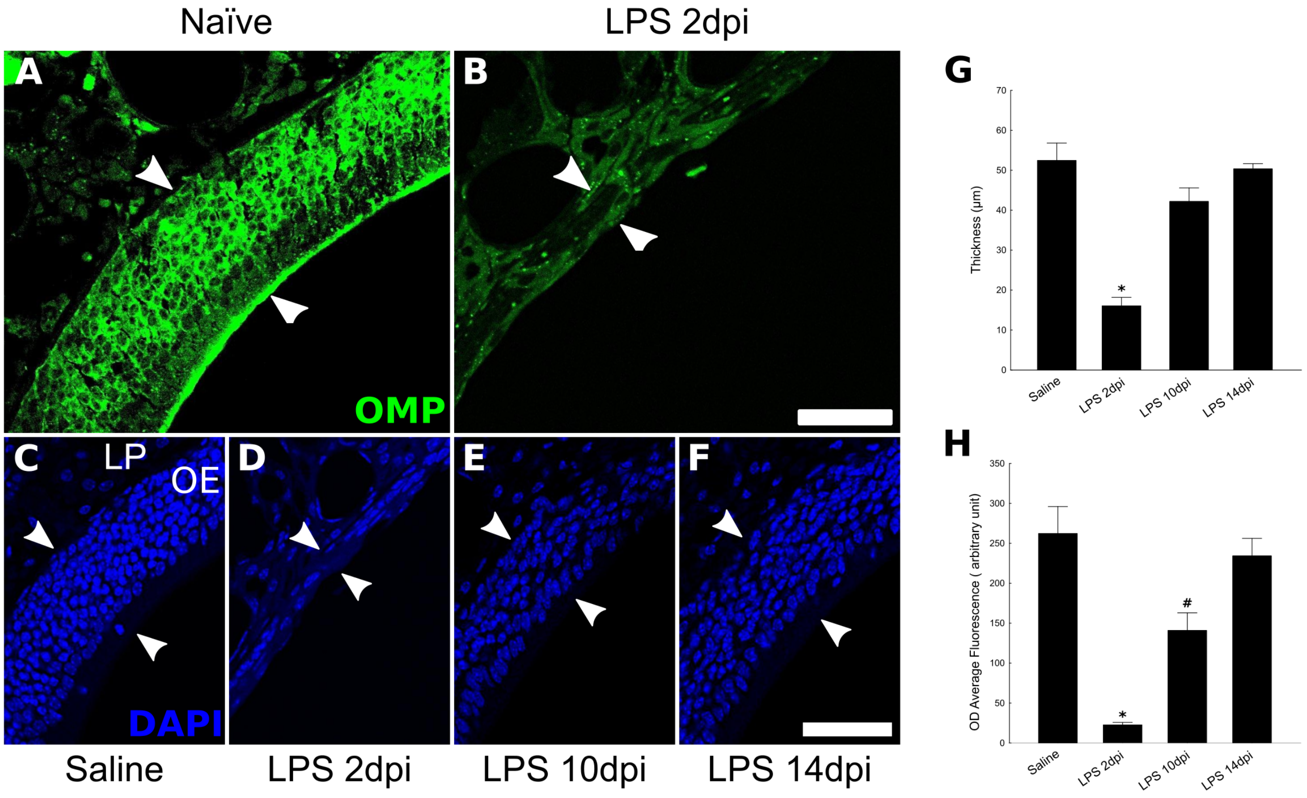

Figure S1 – **Acute intranasal LPS infusion causes reversible lesion of the OE.** (A and B) confocal microscope acquisitions depicting an intact OE and extreme damage provoked by the LPS intranasal infusion as assessed by OMP staining. DAPI staining shows that the TLR4 ligand causes pronounced cell loss of the OE 2 days after the intranasal lesion, which is followed by progressive neuronal recovery is evident at days 10 (E) and 14 (F) post-infusion (dpi). OE regeneration was evaluated by OE cell layer thickness measures (G) and mean optical density from OE sections labeled with the neuronal marker OMP (H). One-way ANOVA [$F(3,8) = 22.5$, $p < 0.0001$ for OD; $F(3,8) = 31.1$, $p < 0.0001$ for OE thickness] followed by a Tukey's HSD multiple comparison test: significantly different (* $p < 0.01$, # $p < 0.05$) from other groups. Please refer to main text for details on experimentation. Arrowheads delimit the OE. Abbreviations: OE, olfactory epithelium; LP: lamina propria. Scale bar: 50 µm.
