# Supplementary material for: Topical Dexamethasone Administration Impairs Protein Synthesis and Neuronal Regeneration in the Olfactory Epithelium
Source: Front Mol Neurosci. 2018 Mar 6;11:50. doi: 10.3389/fnmol.2018.00050 (PMC5845685; doi:10.3389/fnmol.2018.00050)
Supplement: Supplementary file 2 [file Image_2.PDF]

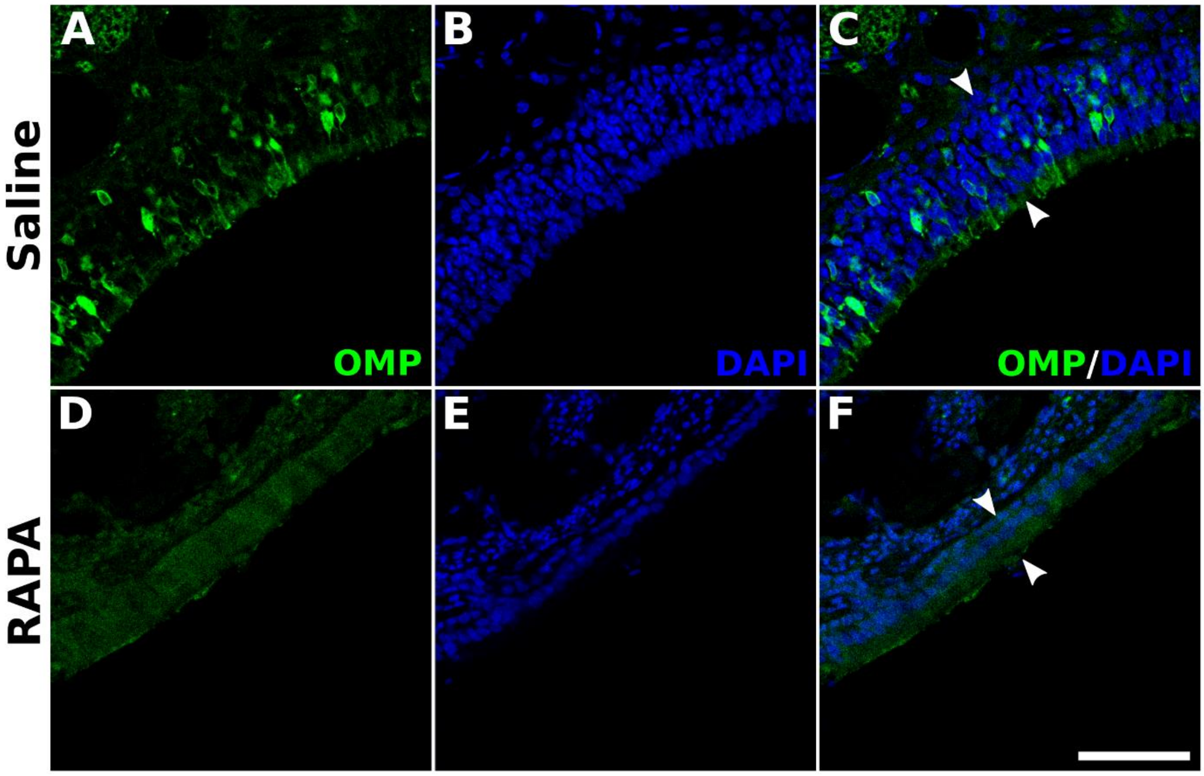


**Figure S2 – Rapamycin impairs OE regeneration after methimazole-induced lesions.** (A - F) confocal microscope acquisitions depicting the effects of control and rapamycin (RAPA) treatments on OE regeneration. The animals received three repeated infusions, once a day, starting from 1 dpl, and were subjected to euthanasia 14 days later. OE sections were processed for OMP (A and D) and DAPI (B and E) staining, revealing a remarkable loss of OSN replenishment due to RAPA treatment. Arrowheads delimits the OE.Scale bar: 50 μm.
